# Biomarkers of response to ocrelizumab in relapsing–remitting multiple sclerosis

**DOI:** 10.3389/fimmu.2024.1480676

**Published:** 2024-11-12

**Authors:** Fernando Rodríguez-Jorge, José Ignacio Fernández-Velasco, Noelia Villarrubia, Julia Gracia-Gil, Eva Fernández, Virginia Meca-Lallana, Carolina Díaz-Pérez, Susana Sainz de la Maza, Eva María Pacheco, Ana Quiroga, Lluis Ramió-Torrentà, Sergio Martínez-Yélamos, Laura Bau, Enric Monreal, Ana López-Real, Alexander Rodero-Romero, Laura Borrega, Santiago Díaz, Pablo Eguía, Mercedes Espiño, Juan Luis Chico-García, Francisco Javier Barrero, María Luisa Martínez-Ginés, José Manuel García-Domínguez, Soraya De la Fuente, Irene Moreno, Raquel Sainz-Amo, M. Alba Mañé-Martínez, Ana Caminero, Fernando Castellanos-Pinedo, Ana Gómez López, Andrés Labiano-Fontcuberta, Lucía Ayuso, Rossana Abreu, Miguel Ángel Hernández, José Meca-Lallana, Lorena Martín-Aguilar, Alfonso Muriel García, Jaime Masjuan, Lucienne Costa-Frossard, Luisa María Villar

**Affiliations:** ^1^ Neurology Department, Hospital Universitario Ramón y Cajal, Madrid, Spain; ^2^ Immunology Department, Hospital Universitario Ramón y Cajal, Madrid, Spain; ^3^ Neurology Department, Complejo Hospitalario Universitario de Albacete, Albacete, Spain; ^4^ Neurology Department, Hospital Universitario La Princesa, Madrid, Spain; ^5^ Neurology Department, Hospital Universitario Juan Ramon Jimenez, Huelva, Spain; ^6^ Neurology Department, Hospital Universitario de Gerona Doctor Josep Trueta, Gerona, Spain; ^7^ Neurology Department, Hospital Universitario de Bellvitge, Barcelona, Spain; ^8^ Neurology Department, Complejo Hospitalario Universitario La Coruña, La Coruña, Spain; ^9^ Neurology Department, Hospital Universitario Fundación Alcorcón, Madrid, Spain; ^10^ Neurology Department, Hospital Universitario Gran Canaria Doctor Negrín, Gran Canaria, Spain; ^11^ Neurology Department, Hospital Universitario Clínico San Cecilio Granada, Granada, Spain; ^12^ Neurology Department, Hospital General Universitario Gregorio Marañon, Madrid, Spain; ^13^ Neurology Department, Hospital Universitario Doce de Octubre, Madrid, Spain; ^14^ Neurology Department, Hospital Universitario Fundación Jiménez Díaz, Madrid, Spain; ^15^ Neurology Department, Hospital Universitario Joan XXII, Tarragona, Spain; ^16^ Neurology Department, Complejo Asistencial de Ávila, Ávila, Spain; ^17^ Neurology Department, Hospital Virgen del Puerto, Cáceres, Spain; ^18^ Neurology Department, Hospital Universitario Principe de Asturias, Madrid, Spain; ^19^ Neurology Department, Hospital Universitario Nuestra Señora Candelaria, Tenerife, Spain; ^20^ Neurology Department, Hospital Clínico Universitario Virgen de la Arrixaca, Murcia, Spain; ^21^ Neurology Department, Hospital de la Santa Creu i Sant Pau, Barcelona, Spain; ^22^ Biostatistics Department, Hospital Universitario Ramón y Cajal, Madrid, Spain

**Keywords:** multiple sclerosis, ocrelizumab, neurofilament light chain, glial fibrillary acidic protein, serum biomarkers

## Abstract

**Objective:**

To ascertain the changes of serum neurofilament light chain (sNfL) and glial fibrillary acidic protein (sGFAP) values in relapsing–remitting multiple sclerosis (RRMS) patients treated with ocrelizumab and their association with treatment response.

**Methods:**

Multicenter prospective study including 115 RRMS patients initiating ocrelizumab treatment between February 2020 and March 2022 followed during a year. Serum samples were collected at baseline and every 3 months to measure sNfL and sGFAP levels using single-molecule array (SIMOA) technology. Based on age and body mass index, sNfL values were standardized using z-score. NEDA (non-evidence of disease activity)-3 status was defined for patients free of disease activity after a year of follow-up. Inflammation (INFL) was considered when new relapses occurred during follow-up or new MRI lesions were found at 1-year exploration. PIRA (progression independent of relapse activity) was defined as disability progression occurring in the absence of relapses or new MRI activity.

**Results:**

After a year on ocrelizumab, 85 patients (73.9%) achieved NEDA-3. Thirty patients did not achieve NEDA: 20 (17.4%) because of INFL and 10 (8.7%) because of PIRA. Of INFL patients, 6 (30.0%) had relapses, and 17 (85.0%) had at least one new MRI lesion at the 12-month examination. At baseline, INFL patients had higher sNfL (p = 0.0003) and sGFAP (p = 0.03) than the NEDA-3 group. PIRA patients mostly exhibited low sNfL and heterogeneous sGFAP levels. After a year, NEDA-3 and INFL patients showed similar decreases in sNfL (p < 0.0001) and sGFAP (p < 0.0001 for NEDA-3 and p = 0.001 for INFL ones). However, the decrease occurred earlier in NEDA-3 patients. Accordingly, sNfL > 1.5 z-score 3 months after ocrelizumab initiation indicated a higher risk of inflammation (OR = 13.6; p < 0.0001). Decrease in sGFAP values occurred later in both groups, with significant reductions observed at 12 months for INFL and 6 and 12 months for NEDA-3. No significant changes in sNfL or sGFAP were observed in PIRA patients.

**Conclusion:**

Ocrelizumab induced normalization of sNfL and sGFAP in the majority of NEDA-3 and inflammatory patients but did not cause changes in the PIRA group. Our data suggest that normalization of sNfL and sGFAP is associated with the lack of inflammatory-associated disease progression but it may not affect non-inflammatory PIRA.

## 1 Introduction

Multiple sclerosis (MS) is a chronic autoimmune disease of the central nervous system (CNS), characterized by inflammation, demyelination, and axonal damage, which leads to the deterioration of neurological function. The accumulation of irreversible disability can result from either relapse-associated worsening (RAW) or progression independent of relapse activity (PIRA) (1). High-efficacy disease-modifying therapies (heDMTs), which include anti-CD20 drugs, have demonstrated near-complete suppression of new relapses and new MRI activity. However, a significant percentage of patients experience silent disability progression (2).

Serum neurofilament light chain (sNfL) levels, obtained within the first year of the disease, have emerged as a useful prognostic biomarker capable of identifying MS patients at high risk of disability progression (3). Early administration of heDMTs in this patient group may prevent such outcomes (4). Conversely, elevated serum glial fibrillary acidic protein (sGFAP) levels appear to be associated with PIRA (5, 6). The impact of heDMTs on patients with high sGFAP values remains to be ascertained.

Ocrelizumab is a humanized monoclonal antibody that specifically targets CD20+ B cells. It has demonstrated a high level of efficacy in preventing new relapses and T2 MRI lesions, with an overall favorable safety profile in clinical trials (7). Furthermore, ocrelizumab has shown a lower progression rate than interferon beta-1a suggesting its potential role in preventing PIRA, particularly in patients with higher exposure to the drug. Ocrelizumab also seems to have a role in decreasing disability progression in older patients than recruited in clinical trials in selected cases under the age of 65 years (8). It has been suggested that higher levels of ocrelizumab in the serum may have a greater impact on disability progression (9). However, there is a lack of predictive factors for clinical response to ocrelizumab in relapsing–remitting MS.

Several studies and authors have explored other biomarkers of response to ocrelizumab in relapsing MS patients, such as switching to a low inflammatory profile increasing T CD8 regulatory cell percentage (10). Kinetics of B-cell repopulation has also shown an influence in radiological activity, being higher in patients with a faster repopulation rate, suggesting a potential benefit of tailoring ocrelizumab dosage in this group (11). In addition, one study has demonstrated that relapsing MS patients switching from fingolimod to ocrelizumab had a suboptimal response compared with those switching from other disease-modifying treatments or naïve patients (12).

Assessing both sNfL and GFAP may be useful for identifying different stages of MS and for predicting prognosis and treatment response (5). Our study aims to evaluate variations of sNfL and sGFAP levels in relapsing–remitting multiple sclerosis (RRMS) patients treated with ocrelizumab and to ascertain the value of both serum biomarkers in predicting response to treatment.

## 2 Materials and methods

### 2.1 Patients

This multicenter prospective longitudinal study was conducted at Ramón y Cajal University Hospital following the Strengthening the Reporting of Observational Studies in Epidemiology (STROBE) reporting guideline. The study involved 115 patients, diagnosed with RRMS according to the revised McDonald criteria (13), who consecutively began ocrelizumab treatment in 20 Spanish university hospitals. Recruitment comprised from February 2020 to March 2022. We collected demographic, clinical, and radiological variables at baseline.

This study received approval from the Ramón y Cajal University Hospital Clinical Research Ethics Committee. Prior to participation, written informed consent was obtained from every patient. Anonymized data supporting the findings of this study will be made available to qualified investigators upon reasonable request for 3 years following the study’s publication.

We followed patients prospectively for a year with clinical assessments every 6 months. Increases of the Expanded Disability Status Scale (EDSS) score were considered based on difference between baseline and 1-year scores. If baseline EDSS was 0, increases were defined for more than 1.5 points. If baseline EDSS was 1 to 5, increases were defined for at least 1 point. If baseline EDSS was >5.5 or greater, increases were defined for 0.5 points. Baseline MRI examination was performed within a month before treatment initiation following clinical protocols established in each center. A second MRI study was performed after a year of follow-up.

We classified patients in three groups according to their response to ocrelizumab treatment after 1 year of follow-up. The NEDA-3 group for patients with no evidence of disease activity: no clinical relapses, no disability progression measured by increases in EDSS score, and no new MRI lesions during the year of follow up. The Inflammatory activity group (INFL) for patients with relapses and/or radiological activity after a year of follow-up, and the PIRA group for patients with disability progression independent of relapses and of new T2 or contrast-enhancing lesions at the 12 month of MRI study. Disability progression was considered when EDSS progression was confirmed 6 months after the first EDSS increase was documented. An additional EDSS evaluation was performed at 18 months from baseline in patients showing disability worsening at 1 year.

### 2.2 Sample collection

Patient blood specimens were obtained just before initiating ocrelizumab treatment and 3, 6, 9, and 12 months thereafter. Serum samples were sent to the Immunology Department of Hospital Ramón y Cajal (Madrid) and stored at −80°C until processed.

### 2.3 Serum NfL and GFAP quantification

sNfL and sGFAP levels were quantified in an SR-X instrument (Quanterix, Lexington, MA) using the single molecule array (SIMOA) technique (Quanterix, Billerica, MA). We used NF-light Advantage Kit (Quanterix, Billerica, MA) and Serum GFAP Discovery Kit (Quanterix, Billerica, MA), respectively, according to the manufacturer’s instructions. A standardized score (z-score), reflecting the age and body mass index-adjusted standard deviations of sNfL levels from a normative data set of healthy controls, was also considered. A value of 1.5 z-score was considered as cut-off based on previous data (3).

### 2.4 Statistical analysis

We performed statistical analyses with GraphPad Prism 9.5 software (GraphPad Prism Inc., San Diego, CA) and Stata v17 (StataCorp LLC). Categorical variables were analyzed with a χ2 or Fisher´s exact test. We used Friedman test adjusted for Dunn multiple comparison test to asses differences between baseline and after 3, 6, 9, and 12 months from the same patients. To evaluate the interaction between times and each patient group, a linear mixed-effects model, multilevel models were used to evaluate time, group, and their interaction, subject as random factor. Kruskal–Wallis was used for inter-group comparisons. Spearman test was used to study correlations between sNfL and sGFAP values. To analyze risks of having a relapse since ocrelizumab initiation, we employed multivariable Cox proportional hazard regressions. These regressions provided hazard ratios (HRs) with corresponding 95% confidence intervals (CIs). The multivariable model was adjusted for age, sex, disease duration, relapses in previous year, baseline EDSS, and T2 lesion load. We considered p-values lower than 0.05 as statistically significant.

### 2.5 Ethical considerations

Written informed consent was obtained from every patient prior to their inclusion in the present study, which was approved by the Ethics Committee of each center participating in this study.

## 3 Results

This multicenter prospective longitudinal study enrolled 115 MS patients [76 women (66.1%), diagnosed at the age of 41.6 ± 10.1 (mean ± SD) years] who initiated ocrelizumab in 20 Spanish hospitals. Twenty-six patients (22.6%) were naïve, and 89 (77.4%) switched from other disease-modifying treatment due to lack of efficacy or safety reasons. Baseline patient data are depicted in **Table 1**.

**Table 1:** Baseline data and patient characteristics.

Variable	All patients (n = 115)
Age (years) Median [range]	39.8 [22.1–65.6]
BMI Median [range]	23.0 [15.4–37.9]
Sex (F/M)	76/39
Current smoker N (%)	12 (10.4%)
Disease duration (years) Median [range]	6.4 [0.1–30.8]
EDSS score Median [range]	2.0 [1.0–6.5]
sNfL Median [range]	7.9 [1.6–382.9]
z-score sNfL Median [range]	0.4 [−3.4–4.0]
sGFAP Median [range]	139.3 [48.6–1,285.1]
Card. Comorbidities N (%)	
High blood pressure Cardiomyopathy Ischemic heart disease	7 (6.1%) 1 (0.9%) 0 (0.0%)
Previous treatment N (%)	
Naïve Platform Orals Monoclonal Ab	27 (23.5%) 27 (23.5%) 43 (37.4%) 18 (15.6%)
Reason for switching previous treatment N (%)	
Effectiveness Safety	78 (88.6%) 10 (11.4%)
T2 lesions on MRI N (%).	
<10 lesions 10–50 lesions 50–100 lesions >100 lesions	12 (10.4%) 77 (67.0%) 23 (20.0%) 3 (2.6%)
Gd lesions on MRI N -%).	
At least one lesion	49 (42.6%)

BMI, body mass index; F, female; M, male; Card, cardiovascular; EDSS, Expanded Disability Status Scale; sNfL, serum neurofilament light chains; sGFAP, serum glial fibrillary acidic protein; n, number of patients; Ab, antibody; Naïve, no previous treatment; Gd: gadolinium-enhancing lesions.

Platform treatments included interferon beta and glatiramer acetate. Oral drugs included cladribine, dimethylfumarate, fingolimod, and teriflunomide. Monoclonal Ab. included alemtuzumab and natalizumab.

After a year on ocrelizumab, 85 patients (73.9%) achieved NEDA-3 status (NEDA-3), 20 (17.4%) had inflammatory activity (INFL), and 10 (8.7%) had disease progression independent of relapses (PIRA). Of the INFL patients, 17 (85.0%) had at least one new T2 lesion at 12-month MRI examination, with 3 (15.0%) of them having new gadolinium-enhancing lesions, and 6 (30.0%) had a relapse during treatment follow-up with a median [range] time of 4.94 [0.10–11.80] months. To analyze the risk of suffering this first relapse since starting ocrelizumab, we performed a multivariable regression analysis incorporating both sNfL z-score and sGFAP levels as predictors (**Table 2**). Patients with elevated sNfL z-scores at baseline exhibit a higher risk of experiencing a relapse (HR of 2.12; 95% CI 1.12–3.99; p = 0.02). sGFAP levels was not associated with heightened risk of relapses.

**Table 2:** Multivariable Cox regression model testing the associations between baseline sNfL z-score and sGFAP levels and the risk of first relapse appearance since o initiation.

	First relapse since OCR	
	HR [95% CI]	p-Value
Age	1.005 [0.914–1.105]	0.919
Sex (male)	0.289 [0.315–2.642]	0.271
Disease duration (years)	1.003 [0.887–1.136]	0.951
Relapses in previous year	0.686 [0.244–1.924]	0.474
Baseline EDSS	0.786 [0.357–1.730]	0.550
Baseline T2 lesion load	0.937 [0.309–2.838]	0.908
Baseline sNfL, z-score	**2.116 [1.119–3.999]**	**0.021**
Baseline sGFAP, pg/ml	0.999 [0.993–1.005]	0.768

Multivariable model was adjusted by age, sex, disease duration, relapses in previous year, baseline EDSS and T2 lesion load. Bold highlight indicates statistically significant values. OCR, ocrelizumab initiation; HR, hazard ratio; CI, confidence interval; EDSS, Expanded Disability Status Scale; sNfL, serum neurofilament light chain; sGFAP, serum glial fibrillary acidic protein.

No baseline differences were found between the three groups in terms of patient age, body mass index, smoking status, cardiovascular comorbidities, disease duration, EDSS score, or number of MRI lesions. However, the percentage of women was higher in the INFL group than that in the NEDA-3 group (87.0% vs. 59.8%, p = 0.02, **Table 3**). Additionally, of no naïve-treatment patients, 78 (88.6%) switched from previous treatment due to lack of effectiveness (appearance of clinical and/or radiological activity) and 10 (11.4%) due to safety reasons, with no significant differences between groups. Interestingly, patients with PIRA received more frequently an oral drug as previous treatment than those in the INFL (p = 0.009, **Table 3**) and NEDA-3 (p = 0.0004, **Table 3**) groups.

**Table 3:** Differences among groups in baseline characteristics.

	NEDA-3 (n = 85)	INFL (n = 20)	PIRA (n = 10)	p-Value NEDA-3 versus INFL	p-Value NEDA-3 versus PIRA	p-Value INFL versus PIRA
Age (years) Median [range]	42.4 [22.1–76.9]	37.3 [25.2–61.4]	44.9 [28.9–62.7]	ns	ns	ns
BMI Median [range]	23.1 [16.7–37.9]	23.8 [18.5–28.8]	21.3 [15.4–29.9]	ns	ns	ns
Sex (F/M)	51/34	18/2	7/3	0.02	ns	ns
Current smoker N (%)	9 (10.6%)	3 (15.0%)	0 (0.0%)	ns	ns	ns
Disease duration Median [range]	7.4 [0.2–28.3]	4.9 [0.1–18.0]	10.7 [1.6–30.8]	ns	ns	ns
EDSS score Median [range]	2.5 [0.0–6.5]	2.0 [1.5–6.0]	3.3 [1.0–6.0]	ns	ns	ns
sNfL Median [range]	7.2 [1.6–120.7]	16.0 [2.2–382.9]	6.7 [2.5–16.7]	0.0006	ns	0.01
z-score sNfL Median [range]	−0.1 [−3.4–3.5]	2.5 [−3.0–4.0]	−0.3 [−3.2–1.0]	0.0003	ns	0.01
sGFAP Median [range]	126.4 [48.6–1,285.1]	199.1 [85.3–1,105.8]	154.5 [75.1–417.2]	0.03	ns	ns
Card. Comorbidities N (%)						
High blood pressure Cardiomyopathy Ischemic heart disease	5 (5.9%) 0 (0.0%) 0 (0.0%)	2 (10.0%) 1 (5.0%) 0 (0.0%)	0 (0.0%) 0 (0.0%) 0 (0.0%)	ns	ns	ns
Previous treatment N (%)						
Naïve Platform Orals Monoclonal Ab.	19 (22.4%) 23 (27.1%) 26 (30.5%) 17 (20.0%)	8 (40.0%) 4 (20.0%) 7 (35.0%) 1 (5.0%)	0 (0%) 0 (0%) 10 (100%) 0 (0%)	ns	0.0004	0.009
Reason for switching previous treatment N (%)						
Effectiveness Safety	58 (87.9%) 8 (12.1%)	11 (91.6%) 1 (8.4%)	9 (90%) 1 (10%)	ns	ns	ns
T2 lesions on MRI N (%).						
<10 lesions 10–50 lesions 50–100 lesions >100 lesions	9 (10.6%) 59 (69.4%) 16 (18.8%) 1 (1.2%)	3 (15.0%) 11 (55.0%) 4 (20.0%) 2 (10.0%)	0 (0.0%) 7 (70.0%) 3 (30.0%) 0 (0.0%)	ns	ns	ns
Gd-lesions on MR N (%).						
At least one lesion	35 (41.2%)	12 (60.0%)	2 (20.0%)	ns	ns	ns

BMI, body mass index; F, female; M, male; Card, cardiovascular; EDSS, Expanded Disability Status Scale; sNfL, serum neurofilament light chains; sGFAP, serum glial fibrillary acidic protein; n, number of patients; NEDA-3, patients free of disease activity at 12 months of ocrelizumab initiation (n = 85); INFL, patients with new relapses and/or radiological activity during follow-up (n =2 0); PIRA, patients who had confirmed disability progression in the absence of relapses or new MRI activity at 12 months of ocrelizumab initiation (n = 10); ns, non-significant; N, number of patients; Ab, antibody; Naïve, no previous treatment; Gd, gadolinium-enhancing lesions.

Platform treatments included interferon beta and glatiramer acetate. Oral drugs included cladribine, dimethylfumarate, fingolimod, and teriflunomide. Monoclonal Ab. included alemtuzumab and natalizumab.

**Figure 1:**
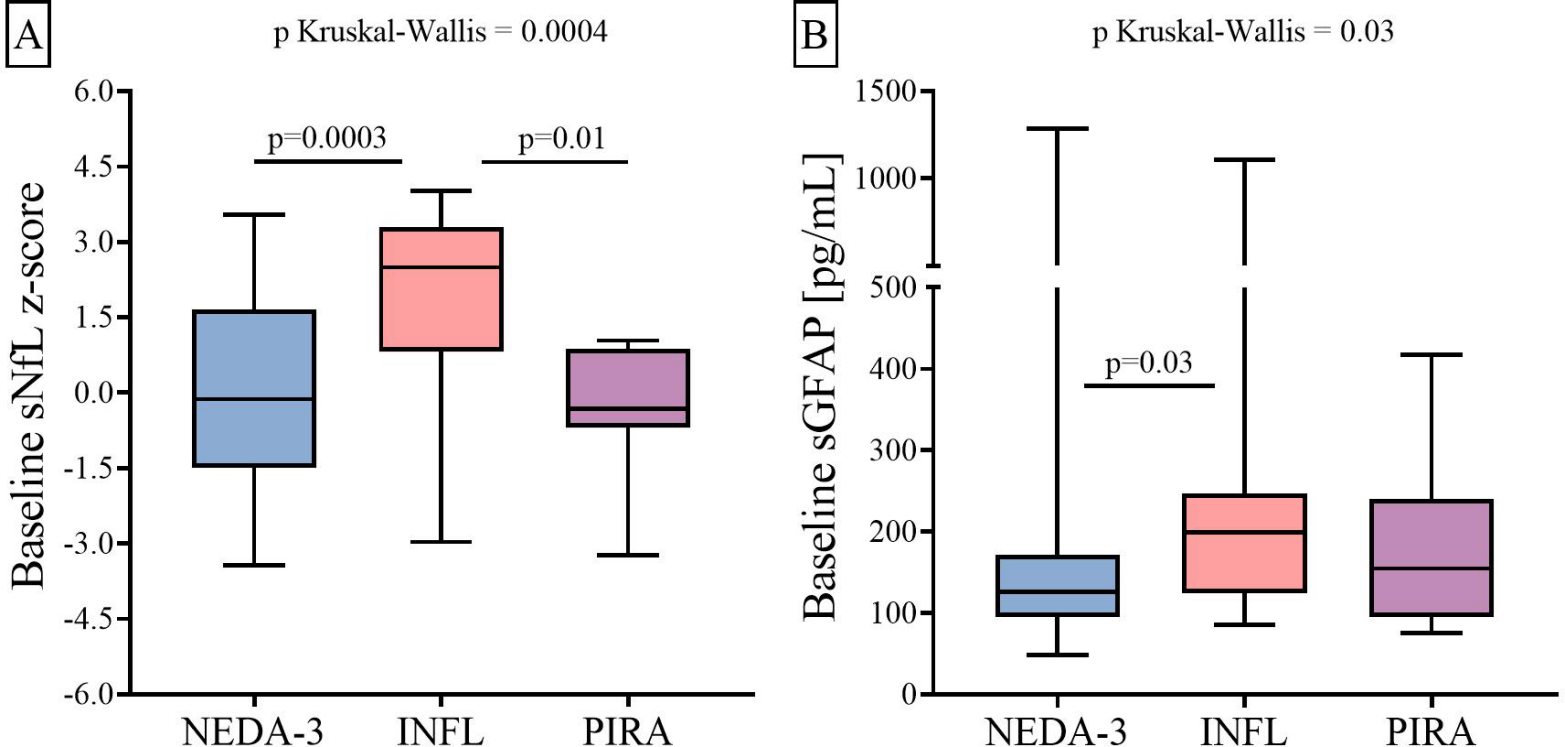
Differences between three groups of RRMS patients in baseline sNfL **(A)** and sGFAP **(B)** levels. RRMS, relapsing–remitting multiple sclerosis; sNfL, serum neurofilament light chains; sGFAP, serum glial fibrillary acidic protein; KW, Kruskal–Wallis test; NEDA-3, patients free of disease activity at 12 months of ocrelizumab initiation (n = 85); INFL, patients with new relapses and/or radiological activity during follow-up (n = 20); PIRA, patients who had confirmed disability progression in the absence of relapses or new MRI activity at 12 months of ocrelizumab initiation (n = 10).

At baseline, the INFL patients exhibited higher sNfL z-score than the NEDA-3 (p = 0.0003) and PIRA (p = 0.01) ones (**Figure 1A**). The INFL group also showed higher sGFAP values than the NEDA-3 group (p = 0.03, **Figure 1B**). By contrast, PIRA patients showed no differences in sGFAP values compared with the other two groups.

**Figure 2:**
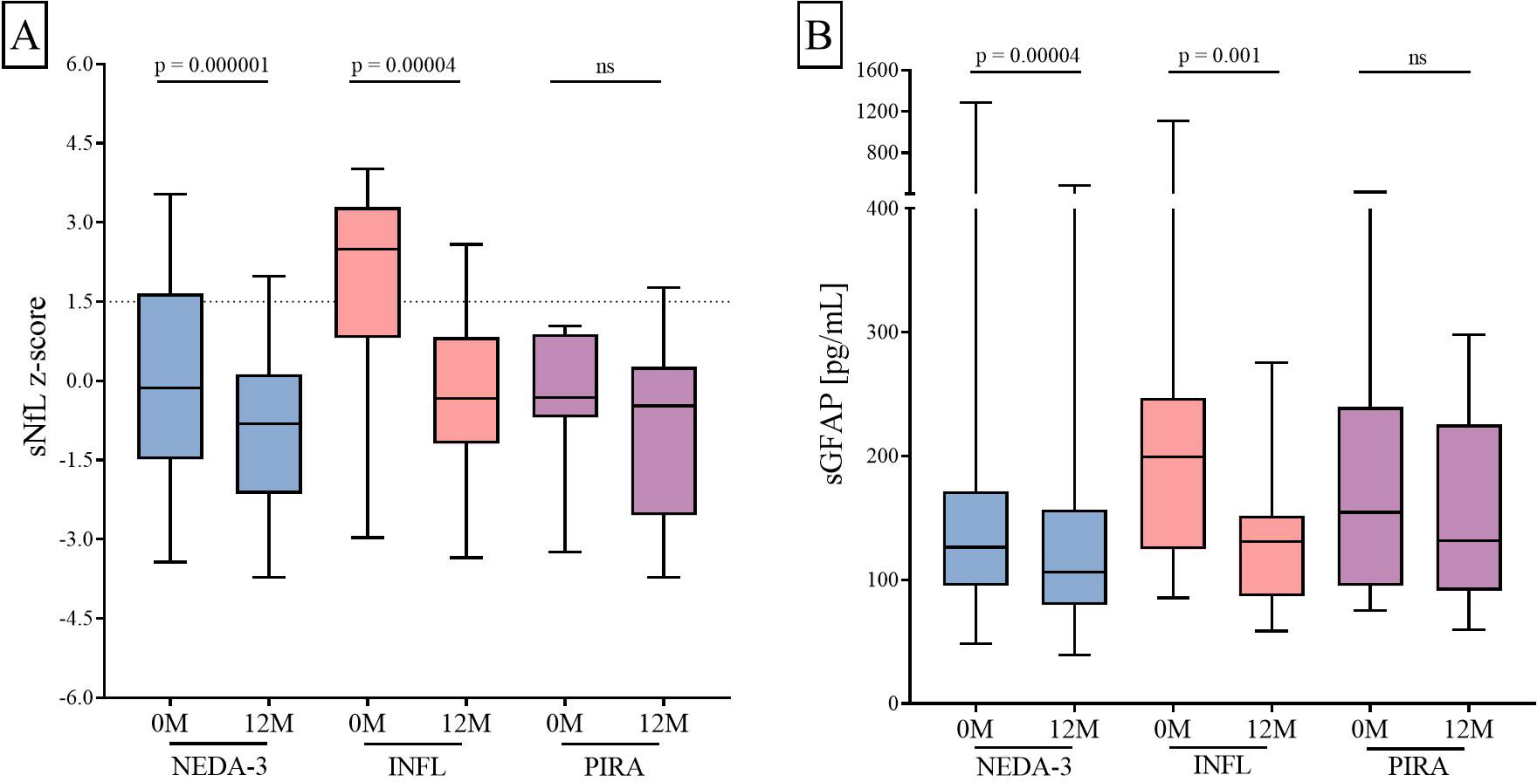
Changes in sNfL **(A)** and sGFAP **(B)** levels induced by ocrelizumab. sNfL and sGFAP levels measured before (0M) and at 12 months (12M) of ocrelizumab initiation. Abbreviations: sNfL, serum neurofilament light chains; sGFAP, serum glial fibrillary acidic protein. ns: non-significant; NEDA-3, patients free of disease activity at 12 months of ocrelizumab initiation (n = 85); INFL, patients with new relapses and/or radiological activity during follow-up (n = 20); PIRA, patients who had confirmed disability progression in the absence of relapses or new MRI activity at 12 months of ocrelizumab initiation (n = 10).

After a year of treatment, sNfL z-score and sGFAP values decreased in the NEDA-3 (p = 0.000001 and p = 0.0004, respectively) and INFL groups (p = 0.00004 and p = 0.001, respectively). However, we did not appreciate significant changes in any of both variables in the PIRA patients (**Figures 2A, B**).

**Figure 3:**
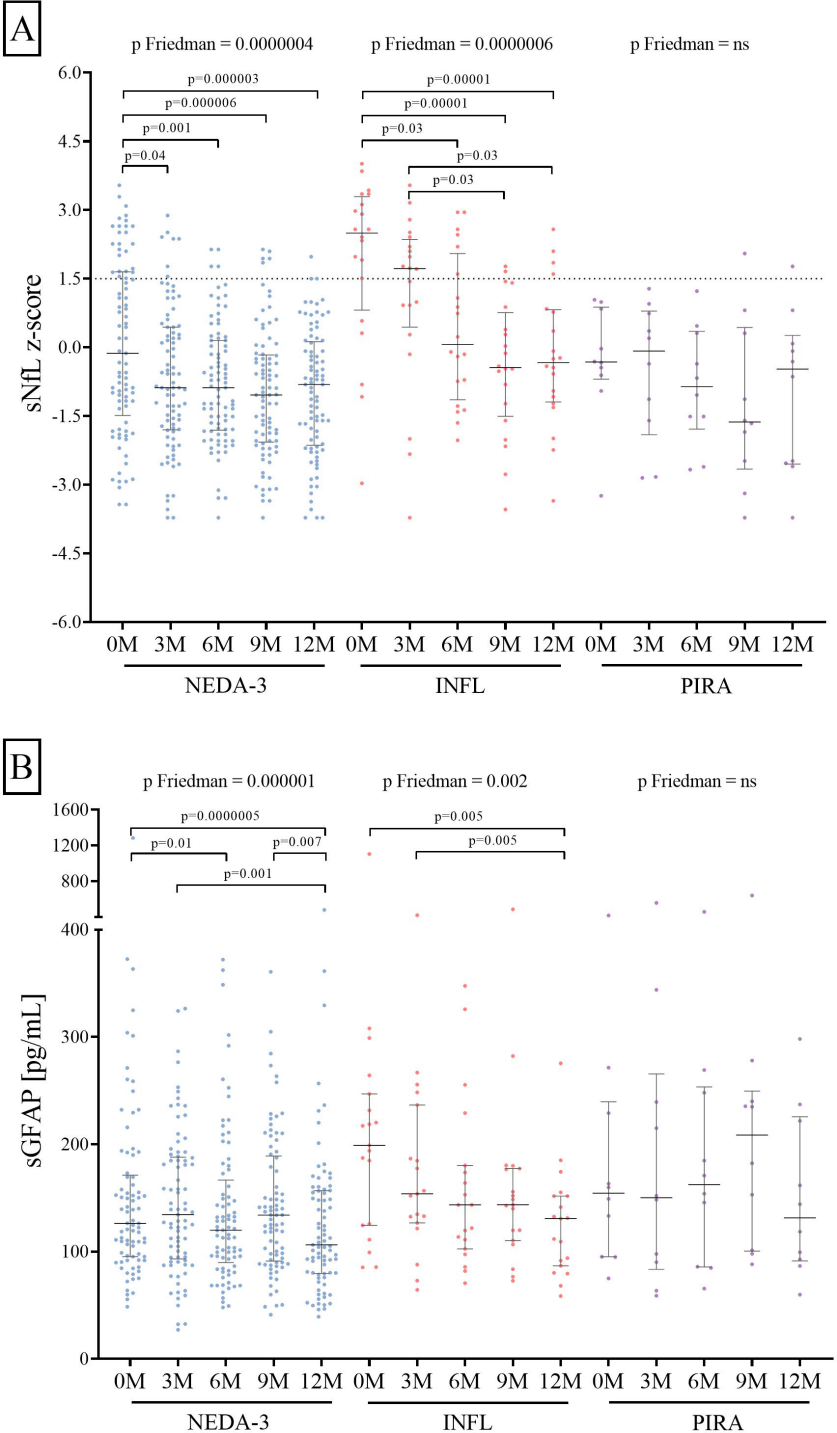
Three-month variations in sNfL **(A)** and sGFAP **(B)** levels induced by ocrelizumab treatment. sNfL and sGFAP levels measured before (0M) and at 3 (3M), 6 (6M), 9 (9M), and 12 (12M) months of ocrelizumab initiation. Abbreviations: sNfL, serum neurofilament light chains; sGFAP, serum glial fibrillary acidic protein; NEDA-3, patients free of disease activity at 12 months of ocrelizumab initiation (n = 85); INFL, patients with new relapses and/or radiological activity during follow-up (n = 20); PIRA, patients who had confirmed disability progression in the absence of relapses or new MRI activity at 12 months of ocrelizumab initiation (n = 10).

We next explored the kinetics of sNfL and sGFAP changes at 3, 6, 9, and 12 months of follow-up (**Figure 3**) where reductions only occurred in both the NEDA (p = 0.0000004 for sNfL z-score and p = 0.000001 for sGFAP) and INFL (p = 0.0000006 for sNfL z-score and p = 0.002 for sGFAP) groups.

While a drop in the sNfL z-score was already observed at 3 months after ocrelizumab initiation in the NEDA-3 group (p = 0.04) and at 6 months in the INFL group (p = 0.03, **Figure 3A**), significant decreases in sGFAP values occurred after 12 months of follow-up. In the NEDA-3 patients, it was observed at 6 months after treatment initiation (p = 0.01) and remained consistent after 1 year (p = 0.0000005, **Figure 3B**). In the inflammatory ones, the reduction became significant at 12 months (p = 0.005, **Figure 3B**). No changes in the sNfL and sGFAP values were observed in the PIRA group.

**Figure 4:**
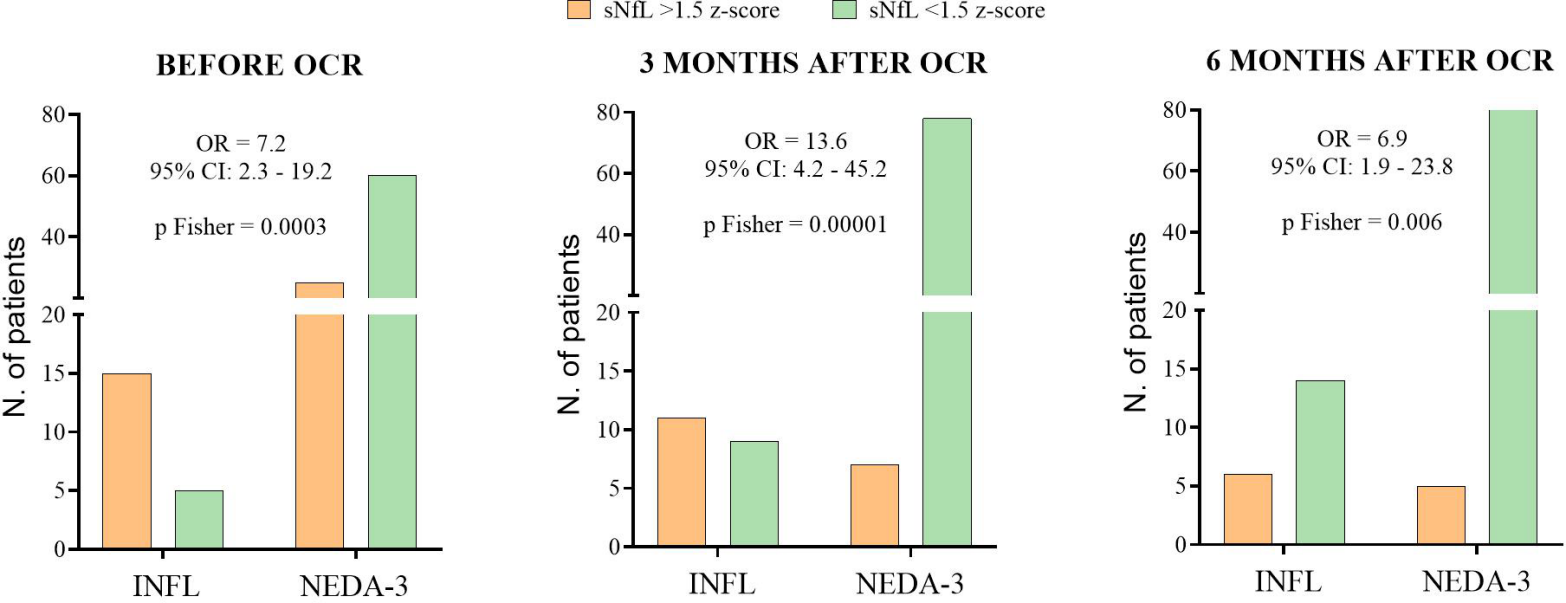
Risk of achieving inflammatory activity at baseline **(A)**, 3 months **(B)**, and 6 months **(C)** of ocrelizumab follow-up. Number (N) of patients with sNFL values above (orange) or below (green) 10 pg/ml. OCR, ocrelizumab treatment; sNfL, serum neurofilament light chains; NEDA-3, patients free of disease activity at 12 months of ocrelizumab initiation (n = 85); INFL, patients with new relapses and/or radiological activity during follow-up (n = 20); OR, odds ratio; CI, confidence interval.

Based on sNfL, we assessed the risk of developing inflammatory activity during the follow-up period (**Figure 4**). A 1.5 sNfL z-score cut-off value was applied, and we analyzed the results obtained from baseline, 3-month, and 6-month samples (**Figures 4A–C**, respectively). At baseline, 70.6% of the NEDA-3 patients had an sNfL z-score below 1.5, whereas only 25.0% of the INFL patients had sNfL at these levels (p = 0.0003). After 3 months of ocrelizumab treatment, 91.8% of NEDA-3 patients achieved an sNfL z-score below 1.5, whereas only 45.0% of INFL patients did (p = 0.00001). The differences remained significant at 6 months (p = 0.006). By 9 months, no significant differences were observed between the two groups. These findings were consistent with the clinical data, as 71.4% of patients who experienced a relapse during treatment did so within the first 6 months following ocrelizumab administration. We selected values obtained at 3 months after initiating ocrelizumab as the most effective predictor (sensitivity 61.1%, specificity 89.7%, negative predictive value 87.4%, **Supplementary Table 1**) of inflammatory status. An sNfL value higher than a 1.5 z-score at this point clearly increased the risk of experiencing inflammatory activity during the first year of treatment [p = 0.00001, odds ratio (OR) = 13.6; 95% confidence interval (CI): 4.2–45.2].

## 4 Discussion

Ocrelizumab is a high-efficacy monoclonal antibody that selectively depletes CD20+ cells while maintaining B-cell reconstitution and pre-existing humoral immunity (14). Different works demonstrated the high efficacy of this drug in RRMS patients (15–17). Different data demonstrated its ability in prevention relapse-associated disability worsening (RAW) as it was observed that most cases of disability progression in patients treated with ocrelizumab were associated with PIRA (18, 19). We aimed to explore the association of sNfL or sGFAP values with RAW and PIRA in a multicenter prospective cohort of 115 RRMS patients treated with ocrelizumab and followed for a year. sNfL levels can serve as a biomarker for monitoring inflammation (3, 20–22) and can predict disease progression (4). Additionally, sGFAP, a marker of astroglial activation, has been associated with MS progression in combination with high sNfL values or as an independent factor (6). High values of sNfL and sGFAP may indicate disease progression associated with acute inflammation, while high sGFAP levels with low sNfL values seem to be more associated with non-inflammatory PIRA (23). However, these biomarkers present some problems for their clinical use. They are not specific for MS and can be increased in other neurological diseases (24) or some other conditions as renal impairment (25). This should be ruled out when evaluating the association of sNfL with MS activity. In addition, sNfL normal values increase with age (3), so it may be difficult to establish the best cut-off values. This was partially solved for sNfL using 10 pg/ml, as the cut off, especially for young individuals, or z-score values (3, 4, 23). For sGFAP, this has been more complicated, but a cut-off value of 140 pg/ml was recently established for individuals younger than 55 years (26).

After follow-up, we classified patients as NEDA-3, INFL, or PIRA according to their response to treatment. sNfL levels reflect acute axonal damage and are strongly associated with acute inflammatory activity manifested as relapses or new MRI lesions (3, 21). Accordingly, we defined inflammatory activity as the presence of new relapses or new T2 or contrast-enhancing MRI lesions. NEDA-3 was described as no inflammatory activity and no disability progression. Different options are being considered to define NEDA-4, with brain atrophy as a widely considered candidate. However, technical issues limit its usefulness, and the use of soluble biomarkers is gaining attention (27). We used sNfL that also reflects axonal damage and is highly reproducible. Additionally, atrophy clearly associates with higher sNfL values (28).

NEDA-3 patients constituted 73.9% of our cohort, the other 17.4% of the patients had inflammatory activity, and the remaining 8.7% experienced PIRA. The NEDA-3 group was characterized by a rapid and maintained decrease in sNfL z-score and a significant decrease in sGFAP values after follow-up. By contrast, inflammatory patients had higher sNfL and sGFAP values at disease onset and showed a more prolonged time to normalize sNfL. In fact, a high sNfL at baseline was associated with a higher risk of relapses during follow-up. By contrast, GFAP levels was not associated with the risk of new relapses. However, after a year, both biomarkers clearly decreased in both groups. This is interesting, since most relapses in the inflammatory group occurred in the first 6 months of treatment, thus corroborating the association of sNfL with acute inflammation (21) and the effect of ocrelizumab in patients with highly inflammatory disease (7, 9, 15). In this line, only a patient with inflammatory activity experienced disability progression showing the effect of ocrelizumab in preventing RAW or inflammatory PIRA (18). Finally, PIRA patients showed low levels of sNfL at treatment onset, but 70% of these patients had high sGFAP values. Ocrelizumab treatment did not change significantly sNfL or sGFAP values in these patients.

Our results can be useful in clinical practice. High sNfL and sGFAP at treatment onset indicate patients at high risk of RAW and inflammatory PIRA, who can respond to treatment with ocrelizumab. Control of the acute inflammatory response seems to precede the normalization of the innate immune response, which has been closely linked to neurodegeneration (29). Conversely, elevated values of sGFAP with normal sNfL may indicate a higher risk of non-inflammatory PIRA. This distinction should aid in fine-tuning treatment selection for MS. In our study, patients with non-inflammatory PIRA had longer disease duration and higher baseline EDSS scores. All of them were previously on oral drugs, and only one had high sNfL levels, compared to the 70% who had elevated sGFAP values unaffected by ocrelizumab treatment. A more inflammatory phase could precede PIRA in these patients, and allowing suboptimal response to treatment for prolonged periods could result in a highly disabling disease with fewer therapeutic options (30). The biomarkers described here could help to prevent therapeutic inertia, which affects up to 25% of daily treatment decisions in MS (31). In fact, early use of ocrelizumab resulted in an optimal response to treatment in naïve-treatment (16) and highly active MS patients (32). Future research is needed to determine the effects of other disease-modifying drugs in preventing both types of disease progression.

Additionally, a high sNfL z-score at the 3-month sample could serve as a reliable biomarker for identifying patients at a high risk of inflammation. This information could be valuable in determining which patients would get profit from a higher dose of ocrelizumab, since the precise characteristics of the patients who would benefit from larger doses are not clearly defined (9).

This study has some limitations such as the relatively limited number of patients and time of follow-up. An independent multicenter study with a higher number of patients followed for a more prolonged period of time would be needed to confirm our data.

In summary, our data demonstrate that sNfL and sGFAP are an important tool to identify patients at risk of inflammatory PIRA, who will benefit from early ocrelizumab treatment. In addition, these biomarkers will contribute, in junction with clinical and MRI data, to avoid therapeutic inertia. It can lead to non-inflammatory PIRA, which reduces the probability of an effective response to treatment in MS patients.

## Data Availability

The raw data supporting the conclusions of this article will be made available by the authors, without undue reservation.

## Supplementary material

Supplementary Table 1 Predictive values of sNFL z-scores in determining inflammation. PPV, positive predictive value; NPV, negative predictive value; M, months; sNfL, sNfL, serum neurofilament light chains.
