# Corrigendum: Biomarkers of response to ocrelizumab in relapsing–remitting multiple sclerosis

**DOI:** 10.3389/fimmu.2024.1535051

**Published:** 2024-12-19

**Authors:** Fernando Rodríguez-Jorge, José Ignacio Fernández-Velasco, Noelia Villarrubia, Julia Gracia-Gil, Eva Fernández, Virginia Meca-Lallana, Carolina Díaz-Pérez, Susana Sainz de la Maza, Eva María Pacheco, Ana Quiroga, Lluis Ramió-Torrentà, Sergio Martínez-Yélamos, Laura Bau, Enric Monreal, Ana López-Real, Alexander Rodero-Romero, Laura Borrega, Santiago Díaz, Pablo Eguía, Mercedes Espiño, Juan Luis Chico-García, Francisco Javier Barrero, María Luisa Martínez-Ginés, José Manuel García-Domínguez, Soraya De la Fuente, Irene Moreno, Raquel Sainz-Amo, M. Alba Mañé-Martínez, Ana Caminero, Fernando Castellanos-Pinedo, Ana Gómez López, Andrés Labiano-Fontcuberta, Lucía Ayuso, Rossana Abreu, Miguel Ángel Hernández, José Meca-Lallana, Lorena Martín-Aguilar, Alfonso Muriel García, Jaime Masjuan, Lucienne Costa-Frossard, Luisa María Villar

**Affiliations:** ^1^ Neurology Department, Hospital Universitario Ramón y Cajal, Madrid, Spain; ^2^ Immunology Department, Hospital Universitario Ramón y Cajal, Madrid, Spain; ^3^ Neurology Department, Complejo Hospitalario Universitario de Albacete, Albacete, Spain; ^4^ Neurology Department, Hospital Universitario La Princesa, Madrid, Spain; ^5^ Neurology Department, Hospital Universitario Juan Ramon Jimenez, Huelva, Spain; ^6^ Neurology Department, Hospital Universitario de Gerona Doctor Josep Trueta, Gerona, Spain; ^7^ Neurology Department, Hospital Universitario de Bellvitge, Barcelona, Spain; ^8^ Neurology Department, Complejo Hospitalario Universitario La Coruña, La Coruña, Spain; ^9^ Neurology Department, Hospital Universitario Fundación Alcorcón, Madrid, Spain; ^10^ Neurology Department, Hospital Universitario Gran Canaria Doctor Negrín, Gran Canaria, Spain; ^11^ Neurology Department, Hospital Universitario Clínico San Cecilio Granada, Granada, Spain; ^12^ Neurology Department, Hospital General Universitario Gregorio Marañon, Madrid, Spain; ^13^ Neurology Department, Hospital Universitario Doce de Octubre, Madrid, Spain; ^14^ Neurology Department, Hospital Universitario Fundación Jiménez Díaz, Madrid, Spain; ^15^ Neurology Department, Hospital Universitario Joan XXII, Tarragona, Spain; ^16^ Neurology Department, Complejo Asistencial de Ávila, Ávila, Spain; ^17^ Neurology Department, Hospital Virgen del Puerto, Cáceres, Spain; ^18^ Neurology Department, Hospital Universitario Principe de Asturias, Madrid, Spain; ^19^ Neurology Department, Hospital Universitario Nuestra Señora Candelaria, Tenerife, Spain; ^20^ Neurology Department, Hospital Clínico Universitario Virgen de la Arrixaca, Murcia, Spain; ^21^ Neurology Department, Hospital de la Santa Creu i Sant Pau, Barcelona, Spain; ^22^ Biostatistics Department, Hospital Universitario Ramón y Cajal, Madrid, Spain

**Keywords:** multiple sclerosis, ocrelizumab, neurofilament light chain, glial fibrillary acidic protein, serum biomarkers

In the published article, an author name was incorrectly written as “Fernando Castellanos”. The correct spelling is “Fernando Castellanos-Pinedo”.

The authors apologize for this error and state that this does not change the scientific conclusions of the article in any way. The original article has been updated.

