# Supplementary material for: Biomarkers of response to ocrelizumab in relapsing–remitting multiple sclerosis
Source: Front Immunol. 2024 Nov 12;15:1480676. doi: 10.3389/fimmu.2024.1480676 (PMC11600310; doi:10.3389/fimmu.2024.1480676)
Supplement: Supplementary Table 1 — Predictive values of sNFL z-scores in determining inflammation. PPV, positive predictive value; NPV, negative predictive value; M, months; sNfL, sNfL, serum neurofilament light chains. [file DataSheet1.pdf]

Supplementary Material

1 Supplementary Figures and Tables

1.1 Supplementary Figures

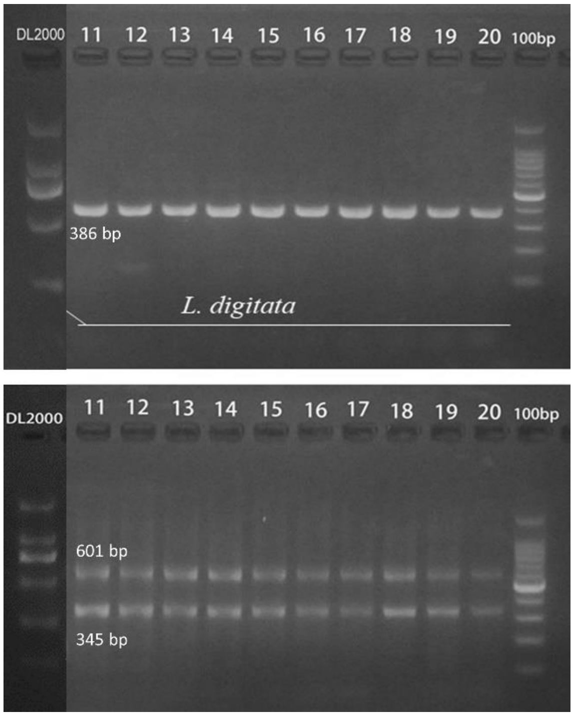

Supplementary Figure 1. For species identification 10 subsamples of the collected specimens were screened. The amplified fragment patterns of PCR1 and PCR2 were compared to those of Mauger et al. (2021). All 10 specimens were identified as *Laminaria digitata* (Hudson) J.V. Lamouroux according to the presence of a band of 386 bp in PCR1 and two bands of 601 bp and 345 bp in PCR2. Samples No. 1–10 were part of another experiment and are therefore not included here.

Mauger, S., Fouqueau, L., Avia, K., Reynes, L., Serrao, E. A., Neiva, J., et al. (2021). Development of tools to rapidly identify cryptic species and characterize their genetic diversity in different European kelp species. *J. Appl. Phycol.* 33, 4169–4186. doi:10.1007/s10811-021-02613-x.

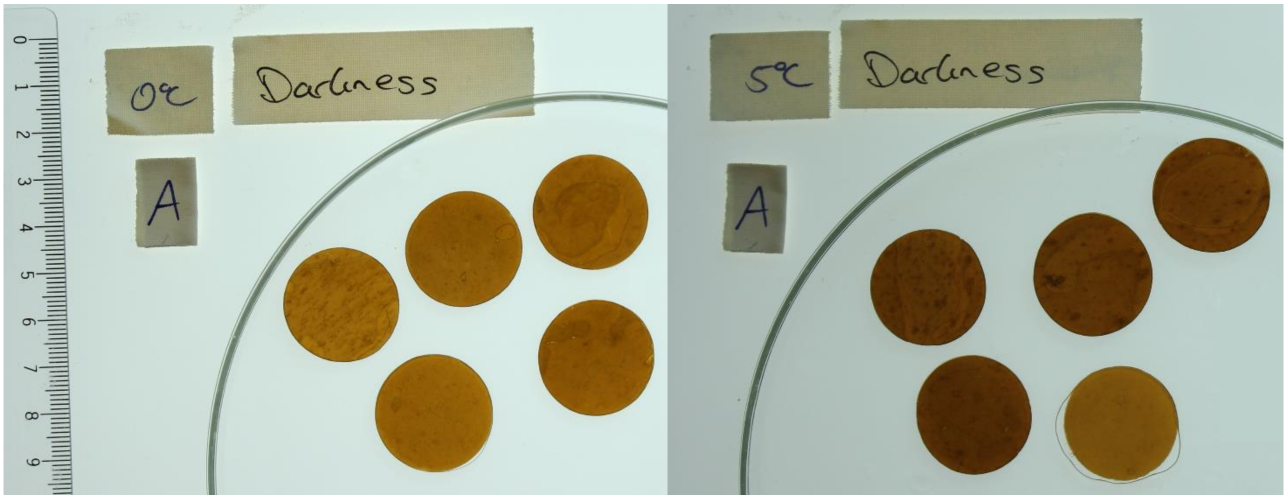

Supplementary Figure 2. Replicate A (n = 6) shown as representative of the condition of all samples after three months of complete darkness.

1.2 Supplementary Tables

F_v/F_m and area of all discs were examined using repeated unifactorial ANOVAs followed by a *post hoc* Tukey test. All other variables, measured in the subsampled discs, were examined using two-factorial ANOVAs followed by *post hoc* Sidak test, due to multiple pairwise comparison.

Supplementary Table S1. Absolute values of disc area, optimum quantum yield of photosynthesis (F_v/F_m), de-epoxidation state of the xanthophyll cycle pigments (DPS) and carbon to nitrogen ratio (C:N) over time. Values are given means ± SD (n = 6). Significances are indicated by asterisks: p < 0.05*, p < 0.01**, p < 0.001***.

Variable	Temperature	Week	Raw data	Direct comparison	
**Area (cm^2)**	0°C	w0	4.911 ± 0.173	0°C = 5°C	w0 = w12
		w2	4.848 ± 0.116		
		w4	4.905 ± 0.175		
		w6	4.849 ± 0.084		
		w8	4.897 ± 0.095		
		w10	4.821 ± 0.064		
		w12	4.942 ± 0.062		
	5°C	w0	4.768 ± 0.037		w0 < w12*
		w2	4.972 ± 0.159		
		w4	4.902 ± 0.141		
		w6	4.825 ± 0.068		
		w8	4.820 ± 0.147		
		w10	4.838 ± 0.051		
		w12	4.907 ± 0.050		
**F_v/F_m (absolute values)**	0°C	w0	0.608 ± 0.023	w0 – w7*** 0°C < 5°C<br><br>w8 – w12*** 0°C > 5°C	w0 = w12
		w1	0.625 ± 0.021		
		w2	0.617 ± 0.022		
		w3	0.632 ± 0.015		
		w4	0.650 ± 0.010		
		w5	0.652 ± 0.010		
		w6	0.638 ± 0.032		
		w7	0.644 ± 0.015		
		w8	0.639 ± 0.013		
		w9	0.675 ± 0.013		
		w10	0.666 ± 0.006		
		w11	0.646 ± 0.009		
		w12	0.625 ± 0.021		
	5°C	w0	0.633 ± 0.021		w0 > w12*
		w1	0.653 ± 0.020		
		w2	0.647 ± 0.027		
		w3	0.654 ± 0.028		
		w4	0.675 ± 0.020		
		w5	0.675 ± 0.021		
		w6	0.662 ± 0.020		
		w7	0.659 ± 0.029		
		w8	0.629 ± 0.028		
		w9	0.650 ± 0.024		
		w10	0.649 ± 0.019		
		w11	0.625 ± 0.028		
		w12	0.614 ± 0.023		

**DPS (absolute values)**	0°C	w0	0.090 ± 0.030	0°C > 5°C***	w0 = w12
		w4	0.046 ± 0.026		
		w8	0.082 ± 0.032		
		w12	0.131 ± 0.063		
	5°C	w0	0.053 ± 0.031		w0 = w12
		w4	0.017 ± 0.007		
		w8	0.036 ± 0.019		
		w12	0.057 ± 0.059		
**C:N (absolute values)**	0°C	w0	21.9 ± 4.1	0°C > 5°C**	w0 = w12
		w4	19.2 ± 4.7		
		w8	19.6 ± 4.7		
		w12	17.9 ± 1.9		
	5°C	w0	20.8 ± 4.7		w0 > w12**
		w4	18.0 ± 4.3		
		w8	13.6 ± 2.4		
		w12	13.4 ± 0.6		

Supplementary Table S2. Statistical details of the monitored variables: F_v/F_m = optimum quantum yield of photosynthesis, Acc = accessory pigments, VAZ = pool of xanthophyll cycle pigments, Chl*a* = chlorophyll *a*, DPS = de-epoxidation state of the xanthophyll cycle, Acc:Chl*a* = ratio of accessory pigments to chlorophyll *a*, VAZ:Chl*a* = ratio of xanthophyll cycle pigments to chlorophyll *a*, C:N = carbon to nitrogen ratio, Total C = total carbon, Total N = total nitrogen. Significances are highlighted in bold.

**Response Variable**	**Factor**		**Df**	**F**	***p*-values**
**F_v/F_m (% of w0)**	Temperature	0°C-5°C	1	142.84	**< 0.0001**
	Sampling		12	40.34	**< 0.0001**
		w1-w0			**0.0414**
		w2-w0			0.9476
		w3-w0			0.1116
		w4-w0			**< 0.0001**
		w5-w0			**< 0.0001**
		w6-w0			**0.0054**
		w7-w0			**0.0033**
		w8-w0			0.8354
		w9-w0			**< 0.0001**
		w10-w0			**< 0.0001**
		w11-w0			0.6324
		w12-w0			1.0000
		w2-w1			0.9993
		w3-w1			0.9999
		w4-w1			0.0796
		w5-w1			0.0503
		w6-w1			0.9256
		w7-w1			0.8745
		w8-w1			0.9999
		w9-w1			0.0643
		w10-w1			0.2828
		w11-w1			1.0000
		w12-w1			0.3387

		w3-w2			0.9402
		w4-w2			**0.0040**
		w5-w2			**0.0022**
		w6-w2			0.3634
		w7-w2			0.2829
		w8-w2			1.0000
		w9-w2			**0.0030**
		w10-w2			**0.0258**
		w11-w2			0.9999
		w12-w2			0.9124
		w4-w3			0.3319
		w5-w3			0.2412
		w6-w3			0.9988
		w7-w3			0.9961
		w8-w3			0.9885
		w9-w3			0.2868
		w10-w3			0.7048
		w11-w3			0.9992
		w12-w3			0.0820
		w5-w4			1.0000
		w6-w4			0.9253
		w7-w4			0.9602
		w8-w4			**0.0115**
		w9-w4			1.0000
		w10-w4			0.9999
		w11-w4			**0.0327**
		w12-w4			< **0.0001**
		w6-w5			0.8612
		w7-w5			0.9160
		w8-w5			**0.0065**
		w9-w5			1.0000
		w10-w5			0.9999
		w11-w5			**0.0196**
		w12-w5			**< 0.0001**
		w7-w6			1.0000
		w8-w6			0.5673
		w9-w6			0.8981
		w10-w6			0.9974
		w11-w6			0.7851
		w12-w6			**0.0035**
		w8-w7			0.4708
		w9-w7			0.9421
		w10-w7			0.9993
		w11-w7			0.6994
		w12-w7			**0.0021**
		w9-w8			**0.0088**
		w10-w7			0.0622
		w11-w7			1.0000
		w12-w7			0.7691
		w10- w9			0.9999
		w11- w9			**0.0258**
		w12- w9			**< 0.0001**
		w11- w10			0.1445
		w12-w10			< **0.0001**
		w12-w11			0.5480

	Temperature:Sampling		12	14.76	< **0.0001**
	Temp. = 0°C	w12-w0			0.9105
		w12- w1			1.0000
		w12-w10			**0.0046**
		w12-w11			0.6313
		w12-w2			0.9999
		w12-w3			0.9999
		w12-w4			0.4062
		w12-w5			0.2770
		w12-w6			0.9715
		w12-w7			0.8026
		w12-w8			0.9772
		w12-w9			**0.0002**
		w0-w1			0.9054
		w0-w10			< **0.0001**
		w0-w11			**0.0135**
		w0-w2			0.9994
		w0-w3			0.4496
		w0-w4			**0.0044**
		w0-w5			**0.0020**
		w0-w6			0.1186
		w0-w7			**0.0314**
		w0-w8			0.1308
		w0-w9			< **0.0001**
		w1-w10			**0.0048**
		w1-w11			0.6412
		w1-w2			0.9999
		w1-w3			0.9999
		w1-w4			0.4156
		w1-w5			0.2847
		w1-w6			0.9737
		w1-w7			0.8104
		w1-w8			0.9790
		w1-w9			**0.0002**
		w10-w11			0.7557
		w10-w2			**0.0003**
		w10-w3			0.0531
		w10-w4			0.9131
		w10-w5			0.9677
		w10-w6			0.2645
		w10-w7			0.5750
		w10-w8			0.2441
		w10-w9			0.9998
		w11-w2			0.1805
		w11-w3			0.9735
		w11-w4			1.0000
		w11-w5			1.0000
		w11-w6			0.9999
		w11-w7			1.0000
		w11-w8			0.9999
		w11-w9			0.2151
		w2-w3			0.9568
		w2-w4			0.0810
		w2-w5			**0.0451**

		w2-w6			0.6358
		w2-w7			0.3120
		w2-w8			0.6634
		w2-w9			< **0.0001**
		w3-w4			0.8858
		w3-w5			0.7776
		w3-w6			1.0000
		w3-w7			0.9956
		w3-w8			1.0000
		w3-w9			**0.0033**
		w4-w5			1.0000
		w4-w6			0.9976
		w4-w7			1.0000
		w4-w8			0.9966
		w4-w9			0.3960
		w5-w6			0.9875
		w5-w7			0.9999
		w5-w8			0.9839
		w5-w9			0.5435
		w6-w7			1.0000
		w6-w8			1.0000
		w6-w9			**0.0306**
		w7-w8			1.0000
		w7-w9			0.1161
		w8-w9			**0.0271**
	Temp. = 5°C	w12-w0			0.8382
		w12- w1			**0.0237**
		w12-w10			0.0620
		w12-w11			0.9977
		w12-w2			0.1180
		w12-w3			**0.0147**
		w12-w4			< **0.0001**
		w12-w5			< **0.0001**
		w12-w6			**0.0010**
		w12-w7			**0.0031**
		w12-w8			0.9762
		w12-w9			0.0558
		w0-w1			0.8433
		w0-w10			0.9573
		w0-w11			0.9999
		w0-w2			0.9898
		w0-w3			0.7601
		w0-w4			**0.0097**
		w0-w5			**0.0091**
		w0-w6			0.2746
		w0-w7			0.4524
		w0-w8			1.0000
		w0-w9			0.9488
		w1-w10			1.0000
		w1-w11			0.3404
		w1-w2			1.0000
		w1-w3			1.0000
		w1-w4			0.6729
		w1-w5			0.6593
		w1-w6			0.9996

		w1-w7			1.0000
		w1-w8			0.5596
		w1-w9			1.0000
		w10-w11			0.5598
		w10-w2			1.0000
		w10-w3			1.0000
		w10-w4			0.4447
		w10-w5			0.4313
		w10-w6			0.9925
		w10-w7			0.9994
		w10-w8			0.7779
		w10-w9			1.0000
		w11-w2			0.7281
		w11-w3			0.2562
		w11-w4			**0.0006**
		w11-w5			**0.0005**
		w11-w6			**0.0409**
		w11-w7			0.0907
		w11-w8			1.0000
		w11-w9			0.5331
		w2-w3			1.0000
		w2-w4			0.2919
		w2-w5			0.2810
		w2-w6			0.9659
		w2-w7			0.9946
		w2-w8			0.8980
		w2-w9			1.0000
		w3-w4			0.7696
		w3-w5			0.7575
		w3-w6			0.9999
		w3-w7			1.0000
		w3-w8			0.4544
		w3-w9			1.0000
		w4-w5			1.0000
		w4-w6			0.9918
		w4-w7			0.9549
		w4-w8			**0.0020**
		w4-w9			0.4706
		w5-w6			0.9905
		w5-w7			0.9503
		w5-w8			**0.0018**
		w5-w9			0.4569
		w6-w7			1.0000
		w6-w8			0.1001
		w6-w9			0.9943
		w7-w8			0.1979
		w7-w9			0.9996
		w8-w9			0.7552
	Sampling = w12	0°C-5°C			**0.007**
	Sampling = w0	0°C-5°C			1.0000
	Sampling = w1	0°C-5°C			0.8868
	Sampling = w10	0°C-5°C			**< 0.0001**
	Sampling = w11	0°C-5°C			**< 0.0001**
	Sampling = w2	0°C-5°C			0.7449

	Sampling = w3	0°C-5°C			0.6552
	Sampling = w4	0°C-5°C			0.8245
	Sampling = w5	0°C-5°C			0.6792
	Sampling = w6	0°C-5°C			0.7091
	Sampling = w7	0°C-5°C			0.2590
	Sampling = w8	0°C-5°C			**0.0007**
	Sampling = w9	0°C-5°C			**< 0.0001**
**F_v/F_m (absolute values)**	Temperature	0°C-5°C	1	142.84	**< 0.0001**
	Sampling		12	40.34	**< 0.0001**
		w1-w0			**< 0.0001**
		w2-w0			**0.0033**
		w3-w0			**< 0.0001**
		w4-w0			**< 0.0001**
		w5-w0			**< 0.0001**
		w6-w0			**< 0.0001**
		w7-w0			**< 0.0001**
		w8-w0			**0.0012**
		w9-w0			**< 0.0001**
		w10-w0			**< 0.0001**
		w11-w0			**0.0072**
		w12-w0			1.0000
		w2-w1			0.3783
		w3-w1			0.9084
		w4-w1			**< 0.0001**
		w5-w1			**< 0.0001**
		w6-w1			**0.0156**
		w7-w1			**0.0026**
		w8-w1			0.9513
		w9-w1			**< 0.0001**
		w10-w1			**< 0.0001**
		w11-w1			0.9999
		w12-w1			**< 0.0001**
		w3-w2			**0.0023**
		w4-w2			**< 0.0001**
		w5-w2			**< 0.0001**
		w6-w2			**< 0.0001**
		w7-w2			**< 0.0001**
		w8-w2			1.0000
		w9-w2			**< 0.0001**
		w10-w2			**< 0.0001**
		w11-w2			0.9988
		w12-w2			0.0576
		w4-w3			**< 0.0001**
		w5-w3			**< 0.0001**
		w6-w3			0.6044
		w7-w3			0.2831
		w8-w3			0.1230
		w9-w3			**< 0.0001**
		w10-w3			**0.0132**
		w11-w3			0.7694
		w12-w3			**< 0.0001**
		w5-w4			1.0000
		w6-w4			**0.0052**
		w7-w4			0.2830
		w8-w4			**< 0.0001**

		w9-w4			1.0000
		w10-w4			0.9952
		w11-w4			**< 0.0001**
		w12-w4			**< 0.0001**
		w6-w5			**0.0047**
		w7-w5			**0.0226**
		w8-w5			**< 0.0001**
		w9-w5			1.0000
		w10-w5			0.9759
		w11-w5			**< 0.0001**
		w12-w5			**< 0.0001**
		w7-w6			1.0000
		w8-w6			**0.0002**
		w9-w6			0.1302
		w10-w6			0.8133
		w11-w6			**0.0320**
		w12-w6			**< 0.0001**
		w8-w7			**< 0.0001**
		w9-w7			0.2902
		w10-w7			0.9512
		w11-w7			**0.0094**
		w12-w7			**< 0.0001**
		w9-w8			**< 0.0001**
		w10-w7			**< 0.0001**
		w11-w7			1.0000
		w12-w7			**0.0199**
		w10- w9			0.9991
		w11- w9			**< 0.0001**
		w12- w9			**< 0.0001**
		w11- w10			**< 0.0001**
		w12-w10			**< 0.0001**
		w12-w11			**0.0293**
	Temperature:Sampling		12	14.76	
	Temp. = 0°C	w12-w0			0.1045
		w12- w1			1.0000
		w12-w10			**< 0.0001**
		w12-w11			0.0724
		w12-w2			0.9714
		w12-w3			0.9806
		w12-w4			**0.0005**
		w12-w5			**0.0003**
		w12-w6			0.5109
		w12-w7			0.0592
		w12-w8			0.4752
		w12-w9			**< 0.0001**
		w0-w1			**0.0010**
		w0-w10			**< 0.0001**
		w0-w11			**< 0.0001**
		w0-w2			0.4983
		w0-w3			**< 0.0001**
		w0-w4			**< 0.0001**
		w0-w5			**< 0.0001**
		w0-w6			**< 0.0001**
		w0-w7			**< 0.0001**

		w0-w8			**< 0.0001**
		w0-w9			**< 0.0001**
		w1-w10			**< 0.0001**
		w1-w11			**0.0066**
		w1-w2			0.7144
		w1-w3			0.7995
		w1-w4			**< 0.0001**
		w1-w5			**< 0.0001**
		w1-w6			0.1003
		w1-w7			**0.0010**
		w1-w8			0.0836
		w1-w9			**< 0.0001**
		w10-w11			0.1302
		w10-w2			**< 0.0001**
		w10-w3			**< 0.0001**
		w10-w4			0.1092
		w10-w5			0.3824
		w10-w6			**< 0.0001**
		w10-w7			**0.0074**
		w10-w8			**0.0001**
		w10-w9			0.9870
		w11-w2			**< 0.0001**
		w11-w3			0.3359
		w11-w4			1.0000
		w11-w5			0.9992
		w11-w6			0.9771
		w11-w7			1.0000
		w11-w8			0.9830
		w11-w9			**0.0015**
		w2-w3			**0.0061**
		w2-w4			**< 0.0001**
		w2-w5			**< 0.0001**
		w2-w6			**0.0001**
		w2-w7			**< 0.0001**
		w2-w8			**0.0001**
		w2-w9			**< 0.0001**
		w3-w4			**0.0006**
		w3-w5			**0.0005**
		w3-w6			0.9764
		w3-w7			0.2724
		w3-w8			0.9660
		w3-w9			**< 0.0001**
		w4-w5			1.0000
		w4-w6			0.3015
		w4-w7			0.9825
		w4-w8			0.3421
		w4-w9			**0.0003**
		w5-w6			0.1768
		w5-w7			0.8985
		w5-w8			0.2034
		w5-w9			**0.0048**
		w6-w7			0.9946
		w6-w8			1.0000
		w6-w9			**< 0.0001**
		w7-w8			0.9967

		w7-w9			**< 0.0001**
		w8-w9			**< 0.0001**
	Temp. = 0°C	w12-w0			**0.0232**
		w12- w1			**< 0.0001**
		w12-w10			**< 0.0001**
		w12-w11			0.9096
		w12-w2			**< 0.0001**
		w12-w3			**< 0.0001**
		w12-w4			**< 0.0001**
		w12-w5			**< 0.0001**
		w12-w6			**< 0.0001**
		w12-w7			**< 0.0001**
		w12-w8			0.3207
		w12-w9			**< 0.0001**
		w0-w1			**0.0001**
		w0-w10			0.1776
		w0-w11			0.9578
		w0-w2			**0.0346**
		w0-w3			**< 0.0001**
		w0-w4			**< 0.0001**
		w0-w5			**< 0.0001**
		w0-w6			**< 0.0001**
		w0-w7			**< 0.0001**
		w0-w8			0.9990
		w0-w9			0.1416
		w1-w10			1.0000
		w1-w11			**0.0001**
		w1-w2			0.9561
		w1-w3			1.0000
		w1-w4			**< 0.0001**
		w1-w5			**< 0.0001**
		w1-w6			0.6460
		w1-w7			0.9581
		w1-w8			**< 0.0001**
		w1-w9			1.0000
		w10-w11			**0.0233**
		w10-w2			1.0000
		w10-w3			0.9992
		w10-w4			**0.0002**
		w10-w5			**0.0007**
		w10-w6			0.6048
		w10-w7			0.8915
		w10-w8			**0.0369**
		w10-w9			1.0000
		w11-w2			**0.0061**
		w11-w3			**< 0.0001**
		w11-w4			**< 0.0001**
		w11-w5			**< 0.0001**
		w11-w6			**< 0.0001**
		w11-w7			**< 0.0001**
		w11-w8			1.0000
		w11-w9			**0.0180**
		w2-w3			0.7577
		w2-w4			**< 0.0001**

		w2-w5			**< 0.0001**
		w2-w6			**0.0317**
		w2-w7			0.1907
		w2-w8			**0.0041**
		w2-w9			1.0000
		w3-w4			**< 0.0001**
		w3-w5			**0.0002**
		w3-w6			0.8888
		w3-w7			0.9974
		w3-w8			**< 0.0001**
		w3-w9			0.9997
		w4-w5			1.0000
		w4-w6			0.1350
		w4-w7			**0.0197**
		w4-w8			**< 0.0001**
		w4-w9			**0.0004**
		w5-w6			0.2205
		w5-w7			**0.0463**
		w5-w8			**< 0.0001**
		w5-w9			**0.0010**
		w6-w7			1.0000
		w6-w8			**< 0.0001**
		w6-w9			0.6670
		w7-w8			**< 0.0001**
		w7-w9			0.9230
		w8-w9			**0.0279**
	Sampling = w12	0°C-5°C			**< 0.0001**
	Sampling = w0	0°C-5°C			**< 0.0001**
	Sampling = w1	0°C-5°C			**< 0.0001**
	Sampling = w10	0°C-5°C			**0.0100**
	Sampling = w11	0°C-5°C			**0.0017**
	Sampling = w2	0°C-5°C			**< 0.0001**
	Sampling = w3	0°C-5°C			**< 0.0001**
	Sampling = w4	0°C-5°C			**< 0.0001**
	Sampling = w5	0°C-5°C			**< 0.0001**
	Sampling = w6	0°C-5°C			**< 0.0001**
	Sampling = w7	0°C-5°C			**0.0015**
	Sampling = w8	0°C-5°C			**0.0469**
	Sampling = w9	0°C-5°C			**0.0002**
**Area (cm^2)**	Temperature	0°C-5°C	1	2.204	0.138
	Sampling		6	9.039	**< 0.0001**
		w2-w0			**0.0002**
		w4-w0			**0.0021**
		w6-w0			0.999
		w8-w0			0.9242
		w10-w0			0.9948
		w12-w0			**< 0.0001**
		w4-w2			0.9978
		w6-w2			**0.0010**
		w8-w2			0.0666
		w10-w2			**0.0029**
		w12-w2			0.6402
		w6-w4			**0.0072**
		w8-w4			0.2210
		w10-w4			**0.0124**

		w12-w4			0.3769
		w8-w6			0.9288
		w10-w6			0.9880
		w12-w6			**0.0001**
		w10-w8			0.7756
		w12-w8			**0.0043**
		w12-w10			**0.0001**
	Temperature:Sampling		6	12.654	< **0.0001**
	Temp. = 0°C	w12-w0			0.9745
		w12-w10			**0.0417**
		w12-w2			0.0682
		w12-w4			0.9370
		w12-w6			0.1102
		w12-w8			0.8758
		w0-w10			0.0749
		w0-w2			0.0837
		w0-w4			1.0000
		w0-w6			0.1811
		w0-w8			0.9980
		w10-w2			0.9792
		w10-w4			0.1192
		w10-w6			0.9823
		w10-w8			0.2761
		w2-w4			0.1569
		w2-w6			1.0000
		w2-w8			0.4641
		w4-w6			0.2883
		w4-w8			0.9999
		w6-w8			0.5958
	Temp. = 5°C	w12-w0			< **0.0001**
		w12-w10			**0.0180**
		w12-w2			0.9990
		w12-w4			0.5171
		w12-w6			**0.0025**
		w12-w8			**0.0014**
		w0-w10			0.4930
		w0-w2			< **0.0001**
		w0-w4			< **0.0001**
		w0-w6			0.2442
		w0-w8			0.3551
		w10-w2			**0.0001**
		w10-w4			0.3059
		w10-w6			1.0000
		w10-w8			1.0000
		w2-w4			**0.0172**
		w2-w6			< **0.0001**
		w2-w8			< **0.0001**
		w4-w6			0.0740
		w4-w8			**0.0417**
		w6-w8			1.0000
	Sampling = w12	0°C-5°C			0.6825
	Sampling = w0	0°C-5°C			< **0.0001**
	Sampling = w10	0°C-5°C			0.8290
	Sampling = w2	0°C-5°C			< **0.0001**

	Sampling = w4	0°C-5°C			0.7996
	Sampling = w6	0°C-5°C			0.4454
	Sampling = w8	0°C-5°C			**0.0077**
**Dry weight (% of w0)**	Temperature	0°C-5°C	1	0.486	0.4899
	Sampling		3	4.555	**0.0077**
		w4-w0			0.1251
		w8-w0			0.0611
		w12-w0			**0.0050**
		w8–w4			0.9869
		w12–w4			0.5625
		w12–w8			0.7643
	Temperature:Sampling		3	0.268	0.8479
	Temp. = 0°C	w12-w0			0.0702
		w12-w4			0.9806
		w12-w8			0.9431
		w0-w4			0.1557
		w0-w8			0.2160
		week-w8			0.9980
	Temp. = 5°C	w12-w0			0.0729
		w12-w4			0.4656
		w12-w8			0.8455
		w0-w4			0.7237
		w0-w8			0.3390
		week-w8			0.9149
	Sampling = w12	0°C-5°C			0.9865
	Sampling = w0	0°C-5°C			1.0000
	Sampling = w4	0°C-5°C			0.2770
	Sampling = w8	0°C-5°C			0.7851
**Mannitol (% of w0)**	Temperature	0°C-5°C	1	1.912	0.174
	Sampling		3	12.694	**< 0.001**
		w0-w12			**< 0.001**
		w4-w12			**0.0019**
		w8-w12			0.1990
		w4-w0			0.2164
		w8-w0			**0.0022**
		w8-w4			0.2490
	Temperature:Sampling		3	0.534	0.1743
	Temp. = 0°C	w12-w0			**0.0082**
		w12-w4			0.0688
		w12-w8			0.8783
		w0-w4			0.8291
		w0-w8			0.0539
		w4-w8			0.2904
	Temp. = 5°C	w12-w0			**< 0.001**
		w12-w4			**0.0241**
		w12-w8			0.1684
		w0-w4			0.2306
		w0-w8			**0.0369**
		w4-w8			0.8175
	Sampling = w12	0°C-5°C			0.1356
	Sampling = w0	0°C-5°C			1.0000
	Sampling = w4	0°C-5°C			0.2860
	Sampling = w8	0°C-5°C			0.8727
**Laminarin (% of w0)**	Temperature	0°C-5°C	1	4.537	**0.0407**
	Sampling		3	5.522	**0.0034**

		w0-w12			**0.0164**
		w4-w12			0.8022
		w8-w12			0.9423
		w4-w0			0.1321
		w8-w0			**0.0036**
		w8-w4			0.4678
	Temperature:Sampling		3	1.386	0.2643
	Temp. = 0°C	w12-w0			0.4145
		w12-w4			0.7241
		w12-w8			0.7476
		w0-w4			0.9550
		w0-w8			0.0682
		w4-w8			0.1905
	Temp. = 5°C	w12-w0			**0.0243**
		w12-w4			0.9988
		w12-w8			0.9861
		w0-w4			**0.0354**
		w0-w8			0.0561
		w4-w8			0.9971
	Sampling = w12	0°C-5°C			0.0920
	Sampling = w0	0°C-5°C			1.0000
	Sampling = w4	0°C-5°C			**0.0173**
	Sampling = w8	0°C-5°C			0.6463
**Chl*a*** **(% of w0)**	Temperature	0°C-5°C	1	2.710	0.1080
	Sampling		3	5.078	**0.0047**
		w0-w12			**0.0354**
		w4-w12			**0.0226**
		w8-w12			0.9651
		w4-w0			0.9975
		w8-w0			0.0800
		w8-w4			0.0520
	Temperature:Sampling		3	0.533	0.6625
	Temp. = 0°C	w12-w0			0.3031
		w12-w4			**0.0486**
		w12-w8			0.9174
		w0-w4			0.7649
		w0-w8			0.6413
		w4-w8			0.1540
	Temp. = 5°C	w12-w0			0.1319
		w12-w4			0.4100
		w12-w8			1.0000
		w0-w4			0.8952
		w0-w8			0.1110
		w4-w8			0.3807
	Sampling = w12	0°C-5°C			0.6584
	Sampling = w0	0°C-5°C			1.0000
	Sampling = w4	0°C-5°C			0.1017
	Sampling = w8	0°C-5°C			0.2619
**Acc** **(% of w0)**	Temperature	0°C-5°C	1	5.321	**0.0269**
	Sampling		3	6.906	**0.0008**
		w0-w12			0.0749
		w4-w12			**0.0062**
		w8-w12			0.9999
		w4-w0			0.6707

		w8-w0			0.0505
		w8-w4			**0.0032**
	Temperature:Sampling		3	0.680	0.5699
	Temp. = 0°C	w12-w0			0.8835
		w12-w4			0.2239
		w12-w8			0.9861
		w0-w4			0.5275
		w0-w8			0.6304
		w4-w8			0.0663
	Temp. = 5°C	w12-w0			**0.0406**
		w12-w4			**0.0289**
		w12-w8			0.9902
		w0-w4			0.9929
		w0-w8			0.0615
		w4-w8			**0.0436**
	Sampling = w12	0°C – 5°C			0.0790
	Sampling = w0	0°C – 5°C			1.0000
	Sampling = w4	0°C – 5°C			0.3090
	Sampling = w8	0°C – 5°C			0.1699
**VAZ (% of w0)**	Temperature	0°C-5°C	1	8.690	**0.0055**
	Sampling		3	32.783	**< 0.0001**
		w0-w12			**< 0.0001**
		w4-w12			**< 0.0001**
		w8-w12			0.9879
		w4-w0			0.4594
		w8-w0			**< 0.0001**
		w8-w4			**< 0.0001**
	Temperature:Sampling		3	0.825	0.4885
	Temp. = 0°C	w12-w0			**0.0008**
		w12-w4			**0.0012**
		w12-w8			1.0000
		w0-w4			0.9990
		w0-w8			**0.0002**
		w4-w8			**0.0003**
	Temp. = 5°C	w12-w0			**< 0.0001**
		w12-w4			**0.0007**
		w12-w8			0.9809
		w0-w4			0.2306
		w0-w8			**< 0.0001**
		w4-w8			**0.0012**
	Sampling = w12	0°C-5°C			0.0951
	Sampling = w0	0°C-5°C			1.0000
	Sampling = w4	0°C-5°C			0.0796
	Sampling = w8	0°C-5°C			0.1098
**DPS (% of w0)**	Temperature	0°C-5°C	1	0.588	0.4481
	Sampling		3	4.014	**0.0146**
		w0-w12			0.5248
		w4-w12			**0.0099**
		w8-w12			0.6348
		w4-w0			0.1554
		w8-w0			0.9974
		w8-w4			0.1070
	Temperature:Sampling		3	0.979	0.4135
	Temp. = 0°C	w12-w0			0.1990
		w12-w4			**0.0146**

		w12-w8			0.2110
		w0-w4			0.5592
		w0-w8			1.0000
		w4-w8			0.5366
	Temp. = 5°C	w12-w0			1.0000
		w12-w4			0.3460
		w12-w8			0.9976
		w0-w4			0.3261
		w0-w8			0.9953
		w4-w8			0.2261
	Sampling = w12	0°C-5°C			0.0637
	Sampling = w0	0°C-5°C			1.0000
	Sampling = w4	0°C-5°C			0.6410
	Sampling = w8	0°C-5°C			0.8435
**DPS (absolute values)**	Temperature	0°C-5°C	1	15.159	**0.0004**
	Sampling		3	5.282	**0.0040**
		w0-w12			0.5572
		w4-w12			**0.0026**
		w8-w12			0.1666
		w4-w0			**0.0462**
		w8-w0			0.8235
		w8-w4			0.2531
	Temperature:Sampling		3	0.742	0.5338
	Temp. = 0°C	w12-w0			0.2940
		w12-w4			**0.0037**
		w12-w8			0.1574
		w0-w4			0.1593
		w0-w8			0.9781
		w4-w8			0.3172
	Temp. = 5°C	w12-w0			0.9980
		w12-w4			0.3084
		w12-w8			0.7723
		w0-w4			0.3583
		w0-w8			0.8439
		w4-w8			0.8160
	Sampling = w12	0°C-5°C			**0.0038**
	Sampling = w0	0°C-5°C			0.0828
	Sampling = w4	0°C-5°C			0.1949
	Sampling = w8	0°C-5°C			**0.0388**
**Acc:Chl*a* (% of w0)**	Temperature	0°C-5°C	1	7.706	**0.0086**
	Sampling		3	3.328	**0.0302**
		w0-w12			0.7663
		w4-w12			0.9998
		w8-w12			0.2560
		w4-w0			0.7808
		w8-w0			**0.0199**
		w8-w4			0.1846
	Temperature:Sampling		3	4.891	**0.0059**
	Temp. = 0°C	w12-w0			**0.0132**
		w12-w4			0.1762
		w12-w8			0.6506
		w0-w4			0.5774
		w0-w8			0.1134
		w4-w8			0.7336

	Temp. = 5°C	w12-w0			0.3768
		w12-w4			0.3627
		w12-w8			**0.0055**
		w0-w4			0.9996
		w0-w8			0.1945
		w4-w8			0.2719
	Sampling = w12	0°C-5°C			**0.0001**
	Sampling = w0	0°C-5°C			1.0000
	Sampling = w4	0°C-5°C			0.2675
	Sampling = w8	0°C-5°C			0.7804
**VAZ:Chl*a* (% of w0)**	Temperature	0°C-5°C	1	8.766	**0.0054**
	Sampling		3	30.787	**< 0.0001**
		w0-w12			**< 0.0001**
		w4-w12			**0.0012**
		w8-w12			0.9744
		w4-w0			*0.0010*
		w8-w0			**< 0.0001**
		w8-w4			**0.0184**
	Temperature:Sampling		3	1.325	0.2817
	Temp. = 0°C	w12-w0			**0.0007**
		w12-w4			0.1243
		w12-w8			0.9991
		w0-w4			0.1169
		w0-w8			**0.0002**
		w4-w8			/
	Temp. = 5°C	w12-w0			**< 0.0001**
		w12-w4			**0.0083**
		w12-w8			0.9922
		w0-w4			**0.0063**
		w0-w8			**< 0.0001**
		w4-w8			**0.0171**
	Sampling = w12	0°C-5°C			**0.0316**
	Sampling = w0	0°C-5°C			1.0000
	Sampling = w4	0°C-5°C			0.1927
	Sampling = w8	0°C-5°C			**0.0255**
**C:N (% of w0)**	Temperature	0°C-5°C	1	4.938	**0.0320**
	Sampling		3	5.557	**0.0028**
		w0-w12			**0.0034**
		w4-w12			0.2862
		w8-w12			0.9712
		w4-w0			0.2435
		w8-w0			**0.0115**
		w8-w4			0.5281
	Temperature:Sampling		3	1.872	0.1497
	Temp. = 0°C	w12-w0			0.3491
		w12-w4			0.9915
		w12-w8			0.9012
		w0-w4			0.5141
		w0-w8			0.7564
		w4-w8			0.9782
	Temp. = 5°C	w12-w0			**0.0051**
		w12-w4			0.1240
		w12-w8			0.9999
		w0-w4			0.5672
		w0-w8			**0.0042**

		w4-w8			0.9782
	Sampling = w12	0°C-5°C			0.0662
	Sampling = w0	0°C-5°C			1.0000
	Sampling = w4	0°C-5°C			0.9317
	Sampling = w8	0°C-5°C			**0.0117**
**C:N (absolute values)**	Temperature	0°C-5°C	1	8.950	**0.0047**
	Sampling		3	5.526	**0.0029**
		w0-w12			**0.0029**
		w4-w12			0.2320
		w8-w12			0.9225
		w4-w0			0.2735
		w8-w0			**0.0163**
		w8-w4			0.5669
	Temperature:Sampling		3	1.285	0.2927
	Temp. = 0°C	w12-w0			0.2567
		w12-w4			0.9325
		w12-w8			0.8595
		w0-w4			0.5845
		w0-w8			0.7019
		w4-w8			0.9975
	Temp. = 5°C	w12-w0			**0.0070**
		w12-w4			0.1603
		w12-w8			0.9996
		w0-w4			0.5574
		w0-w8			**0.0092**
		w4-w8			0.1932
	Sampling = w12	0°C-5°C			**0.0418**
	Sampling = w0	0°C-5°C			0.6038
	Sampling = w4	0°C-5°C			0.5740
	Sampling = w8	0°C-5°C			**0.0080**
**Total C (% of w0)**	Temperature	0°C-5°C	1	0.093	0.7624
	Sampling		3	4.965	**0.0054**
		w0-w12			**0.0108**
		w4-w12			0.0515
		w8-w12			0.9086
		w4-w0			0.9637
		w8-w0			0.0659
		w8-w4			0.2117
	Temperature:Sampling		3	0.472	0.7037
	Temp. = 0°C	w12-w0			0.1481
		w12-w4			0.6070
		w12-w8			0.8246
		w0-w4			0.8924
		w0-w8			0.6209
		w4-w8			0.9757
	Temp. = 5°C	w12-w0			0.0765
		w12-w4			0.0921
		w12-w8			0.9989
		w0-w4			0.9998
		w0-w8			0.1043
		w4-w8			0.1243
	Sampling = w12	0°C-5°C			0.7500
	Sampling = w0	0°C-5°C			1.0000
	Sampling = w4	0°C-5°C			0.5306

	Sampling = w8	0°C-5°C			0.3103
**Total N (% of w0)**	Temperature	0°C-5°C	1	5.387	**0.0258**
	Sampling		3	5.480	**0.0031**
		w0-w12			**0.0049**
		w4-w12			**0.0364**
		w8-w12			0.8014
		w4-w0			0.8998
		w8-w0			0.0600
		w8-w4			0.2557
	Temperature:Sampling		3	1.209	0.3196
	Temp. = 0°C	w12-w0			0.5563
		w12-w4			0.6780
		w12-w8			0.9923
		w0-w4			0.9972
		w0-w8			0.7268
		w4-w8			0.8333
	Temp. = 5°C	w12-w0			**0.0031**
		w12-w4			**0.0319**
		w12-w8			0.7856
		w0-w4			0.8927
		w0-w8			0.0545
		w4-w8			0.2636
	Sampling = w12	0°C-5°C			**0.0195**
	Sampling = w0	0°C-5°C			1.0000
	Sampling = w4	0°C-5°C			0.6050
	Sampling = w8	0°C-5°C			0.1059
